# Urea Glass Route as a Way to Optimize YAGG:Ce^3+^,Cr^3+^,Pr^3+^ Nanocrystals for Persistent Luminescence Applications

**DOI:** 10.1021/acs.langmuir.2c00687

**Published:** 2022-09-13

**Authors:** Vitalii Boiko, Maria Luisa Saladino, Francesco Armetta, Federica Ursi, Marta Markowska, Karina Grzeszkiewicz, Cecilia Mortalò, Cristina Leonelli, Dariusz Hreniak

**Affiliations:** † Institute of Low Temperature and Structure Research, Polish Academy of Sciences, ul. Okólna 2, PL-50-422 Wrocław, Poland; ‡ Department of Biological, Chemical and Pharmaceutical Sciences and Technologies (STEBICEF) and INSTM UdR − Palermo, University of Palermo, Viale delle Scienze, Bld. 17, IT-90128 Palermo, Italy; § Institute of Condensed Matter Chemistry and Energy Technologies (ICMATE), National Research Council of Italy, Corso Stati Uniti, 4, IT-35127 Padova, Italy; ∥ Department of Engineering “Enzo Ferrari”, University of Modena and Reggio Emilia, Via Pietro Vivarelli, 10, IT-41125 Modena, Italy

## Abstract

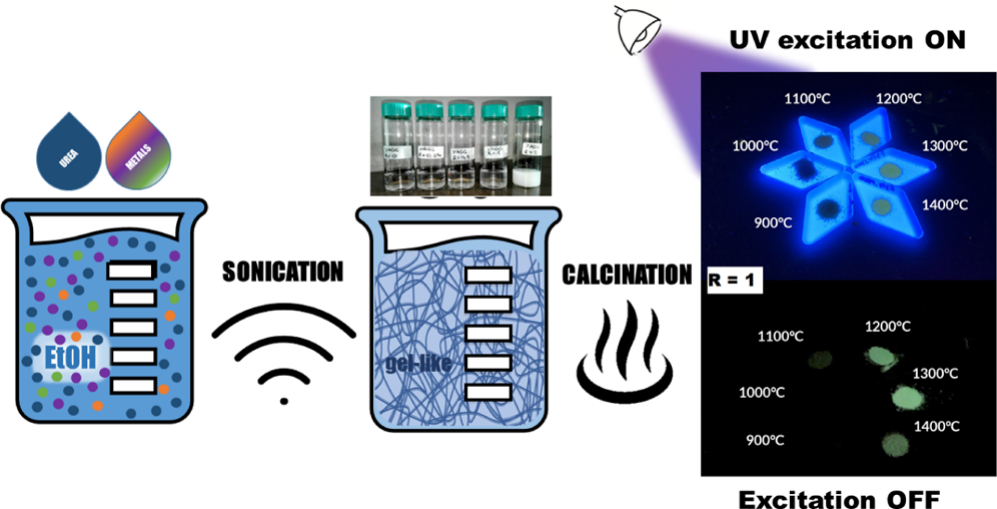

A new approach for the synthesis of Y_3_Al_2_Ga_3_O_12_ (YAGG) nanophosphors allowing the preparation of crystallites with sizes starting from 45 nm is presented. The controllability of the energy and trap density of the resulting material samples by annealing temperature was confirmed by thermoluminescence (TL) measurements. It has been shown that the annealing of samples at temperatures up to 1300 °C does not cause any substantial growth of crystallites, still remaining below 100 nm, but leads to changes in the activation energy of the persistent luminescence (PersL) process. On the other hand, annealing above 1400 °C results in grain growth on the submicron scale, which was confirmed by X-ray powder diffraction (XRPD) and electron transmission microscopy (TEM) measurements. In addition, with an increase in the molar ratio of urea to the total amount of metals used (*R*), qualitative changes are observed in the PersL process occurring from the excited states of Cr^3+^ and Pr^3+^ ions. This proves the influence of the synthesis process, in particular of the metal complexation at its initial stage, on the final structure ordering in the annealed materials. These observations are linked to previously reported defects in the YAGG structure, leading to PersL.

## Introduction

In recent years, great interest in nanomaterials has been observed due to their enormous potential in fundamental studies and the technological field. In this broad class of nanomaterials, an important role is played by luminescent nanoparticles and, in particular, by those activated by trivalent lanthanide ions, due to their promising applications in the fields of bioimaging, lighting, optical nanothermometry, and solar concentrators.^1−3^

Many research groups around the world are strongly motivated to develop not only new hosts for such phosphors but also new synthetic routes to obtain them in the well-defined nanomaterial form.^4−11^ It is well known that the physical properties of crystalline materials are highly dependent on the host material, phase purity, grain size distribution, and crystalline homogeneity. Over the years, mixed oxide systems have been of particular interest due to their stability and design potential for a wide range of properties, such as energy gaps, mainly through ion replacement. In the next step, of course, it is necessary to provide specific properties and the final form of the material, respectively, by appropriate doping and selection and optimization of their synthesis method.^12−14^

**Figure 1:**
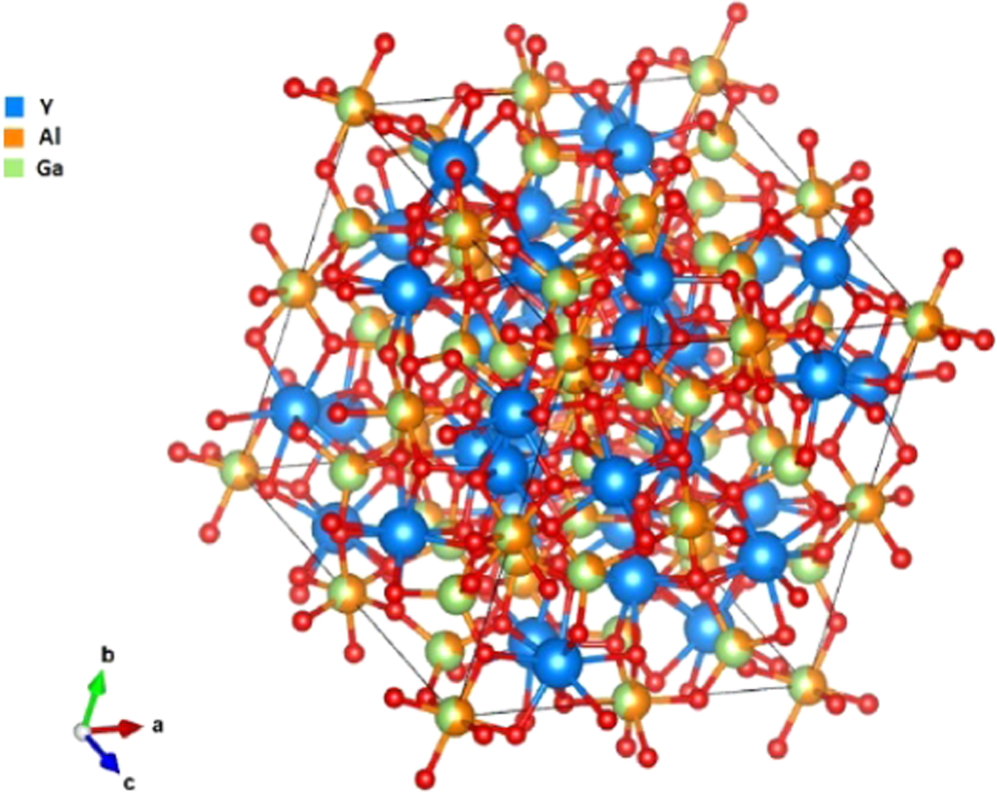
Y_3_Al_2_Ga_3_O_12_ structure.

One of the most promising materials intensively investigated in recent years in various research centers is yttrium aluminum gallium garnet (YAGG, Y_3_Al_2_Ga_3_O_12_), which exhibits specific luminescent properties after appropriate doping.^15,16^ The crystal structure of YAGG is shown in Figure 1. The structure belongs to the space group Ia3̅d. The general chemical formula of an oxide garnet is X_3_A_2_B_3_O_12_, where X refers to a dodecahedral site, A to an octahedral site, and B to a tetrahedral site. The garnet structure has many edges shared between adjacent polyhedra; each tetrahedron and octahedron share edges with two or six dodecahedra, while each dodecahedron divides edges with two tetrahedra, four octahedra, and four other dodecahedra; finally, tetrahedra and octahedra are connected to each other from all angles.^17^ Gilleo and Geller reported that aluminum and gallium prefer tetrahedral sites,^17^ but the reasons are different. Aluminum is located in a four-coordinate site due to the small radius, while the preference of gallium to the tetrahedral site is explained by the nature of Ga^3+^ rather than by the effect of the radius. In fact, the ions with electronic configuration d^10^ tend to settle according to sp^3^ hybridization, preferring the tetrahedral site.^18^ YAGG doped singly or codoped with Cr^3+^ is known as one of the new so-called persistent phosphors, which exhibit continuous luminescence for a long duration (minutes, hours, or days) after ceasing the excitation source.^19−22^ Most recent studies are devoted to nanophosphors emitting in the red and near-infrared (NIR) regions.^23−25^ Although YAGG with such properties could be obtained by its codoping with Cr^3+^ and other trivalent ions, the intensity and duration of persistent luminescence (PersL) in the red region cannot compete with green and blue ones.^19^ PersL in the red region can be used in biomarkers for in vivo imaging, temperature sensing, and security marks in bonds and banknotes.^9,26,27^ Polycrystalline YAGG codoped with Cr^3+^ and Ce^3+^ (YAGG:Ce^3+^,Cr^3+^) has been intensively studied by the Tanabe group.^19,21,28^ The introduction of Cr^3+^ to YAGG:Ce^3+^ was proposed for obtaining long PersL of Ce^3+^ at room temperature. Furthermore, YAGG nanopowders can also be used as a starting material for the production of high-quality optical ceramics with PersL properties, which can be used, for example, in optical storage applications.^29^

The addition of a third dopant to this system allows obtaining new red and near-infrared phosphors, which are of current interest,^30−32^ preferably broad bands excited by blue LEDs. Some doping ions such as Nd^3+^, Pr^3+^, and Yb^3+^ have been successfully used to obtain emissions through efficient energy transfer (ET) from Ce^3+^ and Cr^3+^.^33,34^ In previous works, optimal dopant ratios were already investigated for both spectral properties^35^ and PersL effects.^36^ Special attention has been given to the investigation of the mechanism involved in persistent luminescence,^37−39^ which is correlated to the size and aggregation of particle or lattice defects. Recently, we reported particle size-related limitations of persistent phosphors based on the nanocrystalline YAGG obtained by the Pechini method^30^ and coprecipitation method.^40^ It was found that although at higher annealing temperatures, the properties of the resulting phosphors do not change significantly, the smaller crystallites obtained exhibit more surface defects that can lead to PersL quenching. Therefore, the goal of this study was to investigate the controllability of the energy and trap density of Y_3_Al_2_Ga_3_O_12_:Ce^3+^,Cr^3+^, Pr^3+^ while better maintaining the nanometric size and uniform morphology of particles. To this aim, the urea glass route (UGR) method was chosen for their synthesis, followed by annealing at several temperatures in the range of 900–1400 °C. This one-pot, green, and time-saving synthetic method has already been developed for the preparation of nitride nanoparticles^41^ and Ce:YAG nanopowders^42^ and allows the preparation of materials with high control of powder purity, crystalline structure, and morphology. According to the UGR procedure, metallic salts are solubilized in ethanol, and after the addition of urea, they form a so-called gel-like phase. Urea in ethanol can form complexes with many metals, usually through the carbonyl group or, in the case of more soft metals, with the nitrogen of the amino group. The gel-like phase is the result of the ability of urea to form a polymeric-like organization, which is able to accommodate the nuclei that form during the subsequent heat treatment. Thanks to the formation of an intermediate “gel-/glass-like” material, the primary nanoparticles are stabilized during the heat treatment.

## Experimental Part

### Materials

Urea (Acros Organics, 98%), ethanol (Aldrich, 99.98%), Y(NO_3_)_3_·6H_2_O (Aldrich, 99.8%), Al(NO_3_)_3_·9H_2_O (Aldrich, 98%), Ga(NO_3_)_3_·9H_2_O (Aldrich, 99.9%), Pr_6_O_11_ (Sigma-Aldrich, 99.99%), Ce(NO_3_)_3_·6H_2_O (Aldrich, 99.99%), Cr(NO_3_)_3_·9H_2_O (Fluka 97%) were used in the paper.

### Preparation of Nanocrystals

**Figure 2:**
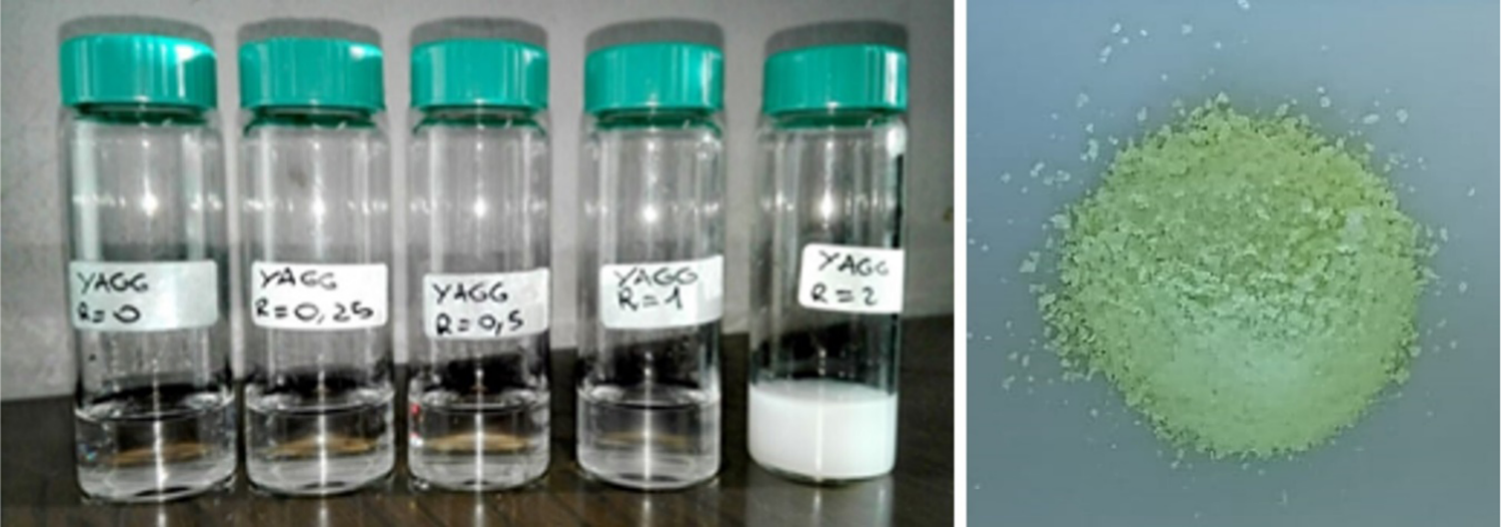
Systems obtained by the urea glass route with urea/metal molar ratios, *R*, of 0, 0.25, 0.5, 1, and 2 (left). Image of the Ce^3+^-, Cr^3+^-, Pr^3+^-codoped powder (right).

YAGG codoped with Ce^3+^, Cr^3+^, and Pr^3+^ nanocrystals were synthesized following this procedure. A solution containing Al(NO_3_)_3_·9H_2_O, Y(NO_3_)_3_·6H_2_O, Ga(NO_3_)_3_·9H_2_O, Pr_6_O_11_, Ce(NO_3_)_3_·6H_2_O, and Cr(NO_3_)_3_·9H_2_O salts in ethanol was prepared in a (Y + Ce + Pr)/(Al + Cr)/Ga molar ratio of 3:2:3 by setting the concentration of Y(NO_3_)_3_ as 0.12 mol L^–1^ to obtain the following formula Y_2.9865_Ce_0.006_Pr_0.0075_Al_1.9875_Cr_0.0125_Ga_3_O_12_. An appropriate amount of urea was added to the ethanolic solution with metals to obtain urea/metal molar ratios, *R*, of 0, 0.25, 0.5, 1, and 2. The obtained systems were subjected to agitation in an ultrasonic bath for 20 min to obtain homogeneous and transparent dispersions identifiable with a gel-like system, as can be observed in Figure 2.

For *R* > 1, the formation of a white dispersion was observed, which separates within a few hours. White dispersion may be associated with the decomposition of urea; for this reason, for the correct purposes of the present paper, only systems with *R* < 1 are suitable. This study was thus performed by calcining only two systems at *R* = 0.25 and 1 for 1 h at temperatures of 900, 1000, 1100, 1200, 1300, and 1400 °C. The system at *R* = 0.5 was not treated and deeply studied because of a few differences in behavior observed between *R* = 0.25 and 1 (see the Results and Discussion section). After the heat treatment, the powders appear green (Figure 2).

### Characterization Techniques

#### X-ray powder diffraction (XRPD)

XRPD patterns were acquired by a Philips PW 1050/39 diffractometer in the Bragg–Brentano geometry using Ni-filtered Cu Kα radiation (λ = 1.54056 Å) in the 2θ range of 5–90° with a step of 0.05° and a time for the step of 5 s. The X-ray generator worked at a power of 40 kV and 30 mA, and the resolution of the instrument (divergent and antiscatter slits of 0.5°) was determined using R-SiO_2_ and R-Al_2_O_3_ standards free from the effect of reduced crystallite size and lattice defects. The phase identification has been performed using X′pert HighScore Software. To obtain information about phase composition, cell parameters, and the crystalline size of the phases, XRPD patterns were analyzed according to the Rietveld method using MAUD software.^43,44^.

#### Transmission Electron Microscopy (TEM)

TEM micrographs of YAGG:Ce^3+^,Cr^3+^,Pr^3+^ nanophosphors annealed at 900, 1000, and 1100 °C were acquired using a JEM-2100 (JEOL, Japan) microscope operating at an accelerating voltage of 200 kV. Each powder was homogeneously dispersed in isopropanol by sonication for 2 min. A drop of the suspension was deposited on a lacey carbon grid of 300 mesh, and after complete solvent evaporation, the grid was introduced into the TEM chamber for analysis. TEM micrographs of YAGG:Ce^3+^,Cr^3+^,Pr^3+^ nanophosphors annealed at 1200 and 1400 °C were acquired using a last-generation high-resolution scanning/transmission electron (S/TEM) microscope (Thermo Scientific Talos F200S) equipped with energy-dispersive X-ray spectroscopy (EDS) and operating at an accelerating voltage of 200 kV. Samples were homogeneously dispersed in bidistilled water (Millipore) by sonication for 10–15 min. A drop of each suspension was deposited on a copper grid of 200 mesh coated with a transparent polymer (Formvar/carbon) and then dried. Subsequently, specimens were carbonated (carbon coater: Balzers CED-010) for TEM investigations.

#### Photoluminescence Emission (PL) and Excitation (PLE) Spectra

Photoluminescence emission (PL) and excitation (PLE) spectra as well as photoluminescence lifetime (microsecond range) were measured using an FLS980 Fluorescence Spectrometer from Edinburgh Instruments. As an excitation source, a 450 W xenon lamp (for PL and PLE) and a 150 W xenon pulse lamp (for a lifetime) were used. An R928P side window photomultiplier tube from Hamamatsu was used as a detector. Both the excitation and emission 300 mm focal length monochromators were in the Czerny Turner configuration. The excitation arm was supplied with a holographic grating of 1800 lines mm^–1^, blazed at 300 nm, while the emission arm was supplied with ruled grating, 1800 lines mm^–1^ blazed at 500 nm. The scanning range was from 250 to 680 nm for PLE spectra and from 460 to 820 nm for PL spectra. For external quantum yield (QY) measurements, an integrating sphere (SM4 from Edinburg Instruments) with a diameter of 30 cm was additionally used. In this case, the signal intensity on the sample was about 60 mW.

The photoluminescence lifetime (nanosecond range) was carried out using a femtosecond laser setup composed of a Coherent Libra-S all-in-one ultrafast oscillator and a regenerative amplifier laser system, with a pulse duration of less than 100 fs at a 1 kHz repetition rate, a Coherent OPerA-Solo optical parametric amplifier, and a Hamamatsu C5677 streak camera with a time resolution of 14 ps.

#### Thermoluminescence (TL)

Thermoluminescence (TL) curves were measured with a lexsygresearch TL/OSL reader (Freiberg Instruments GmbH, Freiberg, Germany). The signal was collected with an R13456 PMT (Hamamatsu Photonics) monitoring the global emission from the whole spectral response (from 185 to 980 nm) with an integration (channel) time of 0.1 s. A blue laser diode PL450B (λ_max_ = 450 nm, FWHM = 2 nm, power 1 mW cm^–2^) by Osram was used as an irradiation source. To learn more about the properties of the traps contributing to the main thermoluminescent peaks, the *T*_max_–*T*_stop_ (partial cleaning) experiment proposed by McKeever was performed.^45^ The method involved the following procedure: the sample was irradiated at room temperature, partially heating the sample to temperature *T*_stop_, cooling to room temperature, and reheating to record (heating rate 0.5 °C s^–1^) all of the remaining glow curves. Positions of the first maximum *T*_max_ in the glow curve versus *T*_stop_ were plotted in the *T*_stop_ range between 30 and 150 °C with a step of 10 °C to cover completely the glow curve. These studies were complemented by an initial rise analysis.^46^ All modules were controlled by LexStudio 2, and the obtained data were processed with Origin software.

## Results and Discussion

### Structure and Morphology

**Figure 3:**
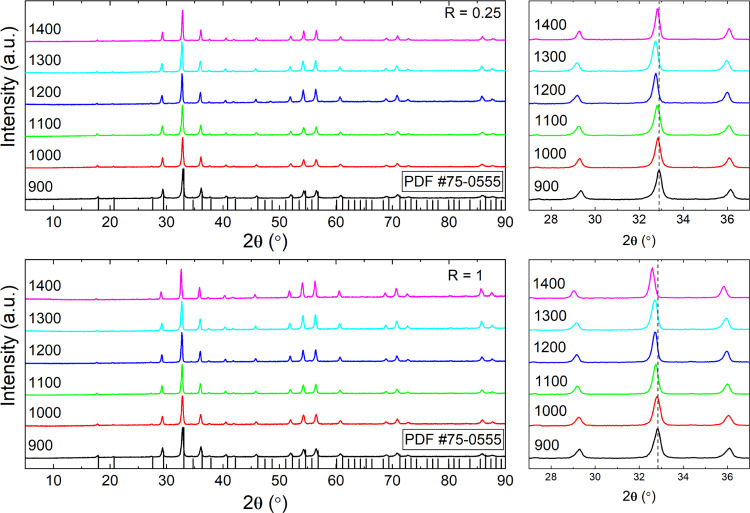
XRD patterns of the YAGG:Ce^3+^,Cr^3+^,Pr^3+^ powder obtained with the UGR method with *R* = 0.25 and 1 calcined in the range of 900–1400 °C. The reference pattern (PDF#75-0555) is reported at the bottom.

The structure of codoped YAGG samples was studied by XRPD. The XRPD patterns of the samples with *R* = 0.25 and 1 treated at various temperatures are shown in Figure 3.

**Figure 4:**
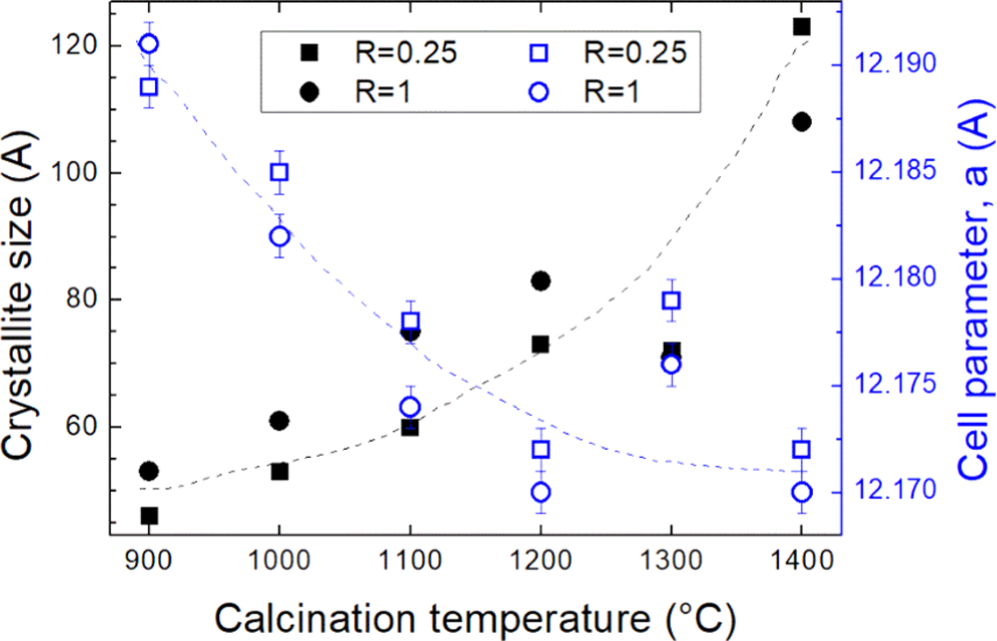
Cell parameter, *a*, and crystallite size, *D*, obtained for YAGG:Ce^3+^,Cr^3+^,Pr^3+^ with *R* = 0.25 and 1 and calcined for 1 h in a temperature range of 900–1400 °C. Dashed lines are a guide for eyes only.

All XRPD patterns are constituted by a single garnet phase^47,48^ and are in good agreement with that of YAGG. The transformation from amorphous to the stable phase garnet occurs completely with heat treatment at a relatively low temperature (900 °C). No additional diffraction peaks were observed, thus indicating the absence of any secondary phases. All diffraction peaks became narrower with increasing temperature, which indicates the higher crystallinity of the powder. This behavior could be attributed to the particle size increase or the strain effect, which could also contribute to the peak broadening of the XRPD patterns. The diffraction patterns were simulated by the Rietveld refinement analysis. For each sample, the agreement between the experiment and the model was evaluated by *R*_b_ (4–6%) and the curve of residues. The cell parameter *a* and the size of crystallites *D* obtained for each sample are given in Figure 4.

The obtained values of the cell parameter *a* are in agreement with the hypothesis of Al^3+^ replacement with Ga^3+^, in agreement with Vegard′s law, and are due to the difference in ionic radii (Ga(III) = 0.76 Å and Al(III) = 0.53 Å).^48^ The cell parameter of undoped YAG is 12.008 Å, while the ones of yttrium gallium garnet (YGG) when all aluminum ions are substituted with gallium is 12.270 Å.^44^ Ga^3+^ ionmainly occupies 24d lattice sites and Al^3+^ occupies 16a lattice sites.^47,48^ The ratio between the octahedral (16a) and tetrahedral (24d) is equal to 2:3. Ce^3+^ and Pr^3+^ ions partially substitute Y^3+^ ions, while Cr^3+^ ions replace aluminum ions in the octahedral position.^49^

As the analysis of the diffraction patterns of the two series showed no significant differences between the cell parameter, *a*, and the crystallite size, *D*, subsequent studies thus focused on the effect of temperature. During the heat treatment, urea has been decomposed releasing gaseous products that do not interfere with the formation of the garnet phase but result in possible defects or in formation of small particles. In both cases, *R* = 0.25 and 1, the decrease in the cell parameter with increasing annealing temperature can depend on a homogeneous disorder introduced into the lattice during the amorphous to crystalline transformation and on an imperfect local stoichiometry, which causes an increase in the lattice parameter.

On the other hand, the treatment at a higher temperature allows the release of this disorder with further densification even if in both series the microstrain ε was between 1 × 10^–5^ and 4 × 10^–5^, without a significant effect from the temperature. The average crystallite size increases by increasing the temperature for both series, as already observed.^30,40^

**Figure 5:**
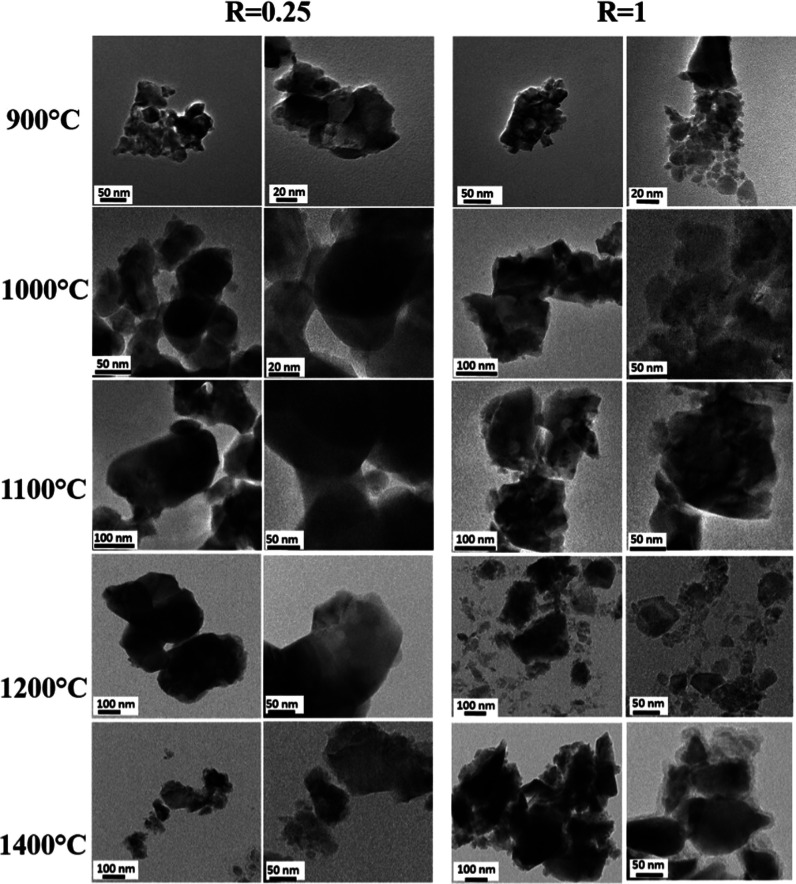
TEM micrographs at different magnifications of YAGG:Ce^3+^,Cr^3+^,Pr^3+^ powders.

Some representative TEM micrographs with different magnifications of the YAGG:Ce^3+^, Cr^3+^, Pr^3+^ samples prepared at *R* = 0.25 and 1 after annealing in the range of 900–1400 °C are reported in Figure 5 and in Figure S1 with lower magnification. Two different enlargements have been chosen per sample to evidence primary nanoparticle distribution (low enlargements) and the details of each particle or aggregate (high enlargements).

TEM micrographs clearly show that the treatment at 900 °C causes the formation of agglomerates made of few YAGG particles not regular in shape. Particles of smaller size and more uniform in shape are formed starting from being gel-like at *R* = 1. Similar results were obtained for Ce:YAG nanocrystals in a previous study of some of us, where *R* = 1 was considered the best to obtain a more homogeneous material.^42^ The annealing process at a higher temperature sharpens the nanoparticles′ edges and evidences their irregular polyhedral shapes for all temperatures. The particle size increases with the temperature, as well as the crystalline size evaluated by XRPD data. However, the particle size is a little bit bigger than the crystalline size, and this can be explained considering that TEM permits to observe the details of the particles (with a polydispersion), while the XRPD technique evaluates the material in all volumes, giving an average of the crystalline size. The effect of *R* is not so significant in the mean value of particle size. The crystalline nature of the particles has been evident in collecting the SAED patterns (reported in Figure S2a). All elements (Ga, Al, Y, Cr, Ce, Pr) are present in the EDS spectra (Figure S2b), and their ratio corresponds to the ones of the nominal composition.

### Photoluminescence

To correlate the crystal structure parameters of the samples obtained at different synthesis conditions with the local structure of the dopant ions, which have a fundamental influence on their luminescence properties, selective studies by optical spectroscopy were carried out in the next step. In addition to the primary mechanism leading to PersL, which is the direct relaxation of the trapped energy due to irradiation into the luminescent ion, it is also possible to observe PersL resulting by exploiting in some cases the more likely energy transfer to the same ion through other dopants present in the material. Such an extended process to obtain a PersL signal can be studied in detail by analyzing the energy transfers, leading to normal photoluminescence occurring along, exactly, the same pathway. It is, therefore, crucial to gather basic information about possible energy transfers with the help of the analysis of emission, excitation, and lifetime spectra of subsequent excited levels involved in these transfers.

**Figure 6:**
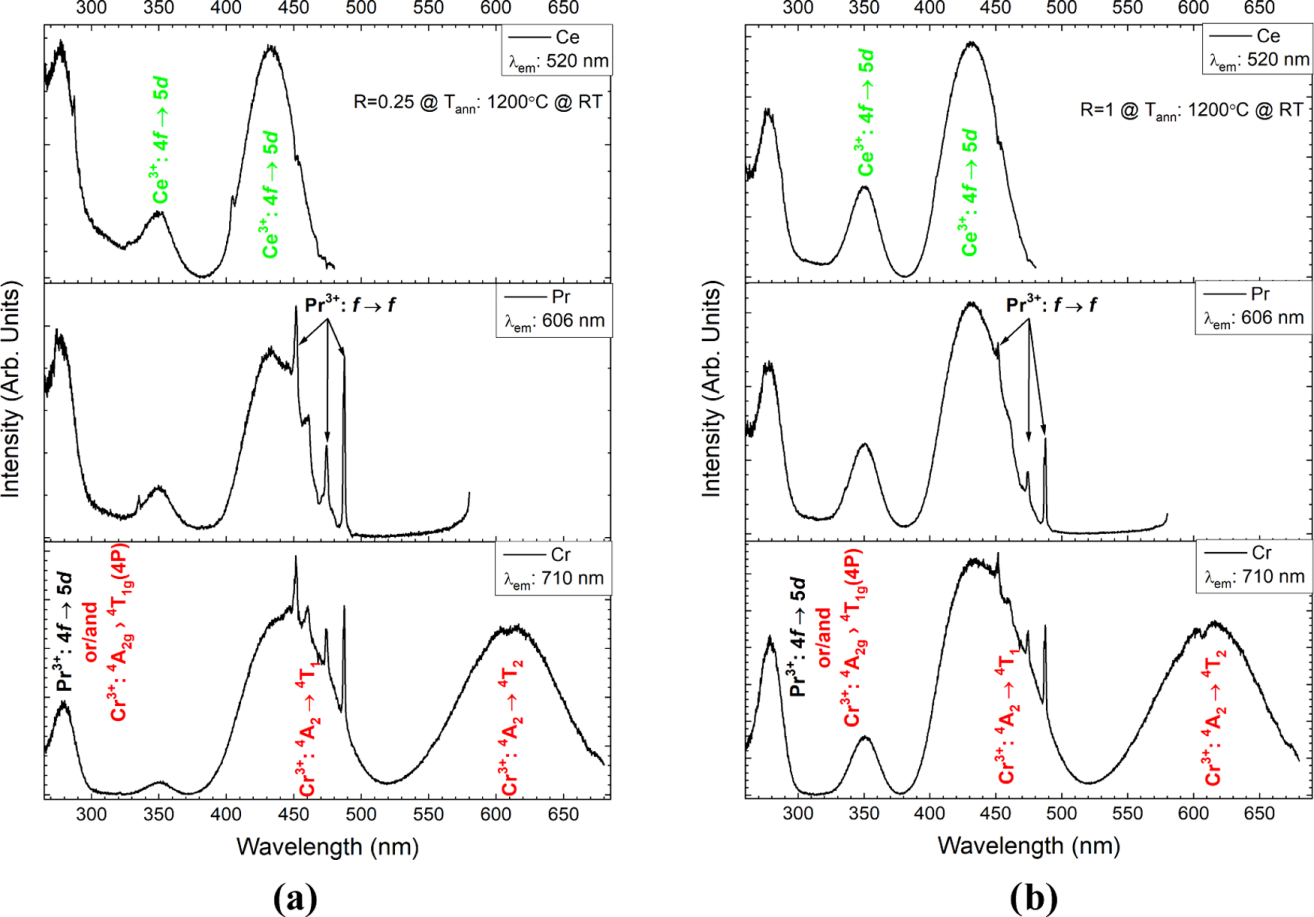
Photoluminescence excitation spectra of the YAGG:Ce^3+^,Cr^3+^,Pr^3+^ powder synthesized at *R* = 0.25 (a) and 1 (b) and calcined at 1200 °C.

The PLE spectra of the samples for *R* = 0.25 and 1 are presented in Figures 6 and S3. The PLE spectra were acquired at λ_em_ = 520, 606, and 710 nm (respectively for well-known Ce^3+^, Pr^3+^, and Cr^3+^ emission peaks). All collected spectra show broad bands centered at 280, 350, and 435 nm, and a series of sharp lines at ∼450 nm additionally appeared in the PLE spectra of Pr^3+^ and Cr^3+^. A detailed analysis of energy transfer based on PLE spectra for this dopant system was carried out in our previous work.^40^ Briefly, the band centered at 280 nm is assigned to Pr^3+^:4f → 5d transition^50^ and most likely the overlapping Cr^3+^:^4^A_2g_ → ^4^T_1g_(4P) transition.^37^ The presence of this band also in the PLE spectrum of Ce^3+^ indicates a pronounced ET from the lowest-lying 4f5d state of Pr^3+^ to the 5d state of Ce^3+^.^51^ In the excitation spectra of Ce^3+^ emission (520 nm), the two bands centered at 350 and 435 nm are attributed to the well-known splitting of the 4f → 5d Ce^3+^ state by the crystal field.^52^ In the excitation spectra of both Pr^3+^ (^1^D_2_ → ^3^H_4_, λ_em_ = 606 nm) and Cr^3+^ (^4^T_2_ → ^4^A_2_, λ_em_ = 710 nm), characteristic bands in the blue light range associated with f–f transitions of Pr^3+^ were recorded. Additionally, in the PLE spectrum of Cr^3+^, a typical ^4^A_2_ → ^4^T_2_ transition in the red range with a maximum at 617 nm was found.

**Figure 7:**
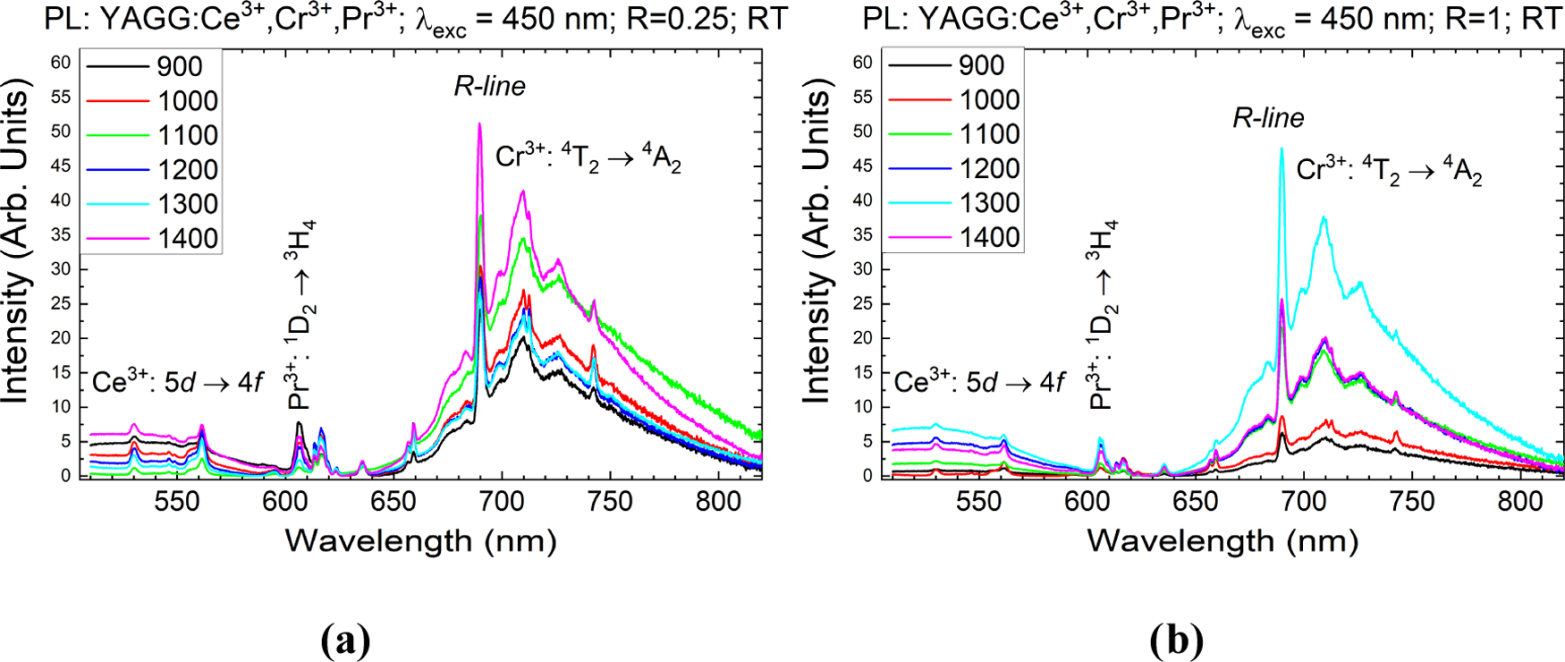
Photoluminescence spectra of the YAGG:Ce^3+^,Cr^3+^,Pr^3+^ powder synthesized at *R* = 0.25 (a) and 1 (b). λ_exc_ = 450 nm at RT.

The PL spectra acquired upon 350 nm excitation and presented in Figure S4 show a characteristic broad band corresponding to the d–f transition of Ce^3+^ with a maximum centered at 512 nm and a sharper band with a maximum at 712 nm (Cr^3+^:^4^T_2_ → ^4^A_2_ transition). In between, less intensive but evident narrow lines are also present due to indirectly induced f–f transitions of Pr^3+^:^1^D_2_ → ^3^H_4_ (606 nm) and ^3^P_0_ → ^3^H_6_ (634 nm) evident upon 450 nm excitation (Figure 7).

In terms of practical application, the observed spectral characteristics and the possibility of obtaining efficient emission from all ions upon 450 nm excitation (Figure 7), which can be generated using commonly available LEDs, are very advantageous.^53,54^ In general, an increase in the intensity of all bands was noticed as the annealing temperature increased for both *R* = 0.25 and 1 samples. This behavior is a consequence of the fact that the surface-to-volume ratio decreases together with the decrease of the probability of nonradiative recombination through the elimination of quenching defects.^55,56^ To find out which of the UGR synthesis parameters yields better results, affecting the final properties of PersL, it was necessary to study these aspects in more detail by analyzing the luminescence kinetics.

The luminescence lifetime is a very important parameter for understanding the quenching mechanisms related to many aspects of nonradiative relaxation of excited states depending mainly, but not exclusively, on the distance between dopant ions (their concentration), the local crystal field of the luminescence center and its distortions, grain size, effective refractive index, and the presence of the OH group.^55−57^ On the other hand, it provides with the combined analysis of the excitation spectra an understanding of the excitation pathways between dopant ions. In view of this, the lifetimes for emission at 520 nm (Ce^3+^:5d → 4f transition), 606 nm (Pr^3+^:^1^D_2_ → 3H4 transition), and 690 nm (Cr^3+^:^4^T_2_ → ^4^A_2_ transition) after excitation at 350 nm (specific for Ce^3+^) and 450 nm for exciting all ions at the same time were detected (Figures S5 and S6, respectively). Then, an effective (average) lifetime was calculated (Figure S7). From the profile of the lifetime Ce^3+^ emission, it can be seen that increasing annealing temperatures has a slight impact on decreasing lifetime, which is related to crystalline size and has no significant effect on the energy transfer to Pr^3+^ and Cr^3+^. The average lifetime for Pr^3+^ coincides with the values previously obtained for the garnet matrix^58^ and confirms the affiliation of this band to the ^1^D_2_ → ^3^H_4_ transition. At the same time, it is worth noting that the lifetime may depend on many factors, and as we can see at annealing temperatures from 900 to 1100 °C, the number of hydroxyl groups,^30^ which are the quenching centers, decreases but at the same time the so-called effective refractive index, which affects the rate of radiation processes from lanthanide ions, increases. As a result, depending on the contribution of both factors, one can observe first an increase in the lifetime with quenching and then its decrease to values typical for reference single crystals. The effect of the effective refractive index, which can also be related to the degree of agglomeration (in the case of powders) as well as to porosity (of optical ceramics) on the rate of radiation processes in luminescent materials, is quite well described in previous papers.^55,57^ What is relevant here is only the information that with the increasing agglomeration of powders (or removal of porosity from ceramics) there is a shortening of the measured lifetime, finally to a value corresponding to the lifetime of the monocrystals of the compounds under consideration.

The QYs for all samples were detected after excitation at both 350 and 450 nm with an integrated sphere, and the obtained results are reported in Figures S8 and 8.

**Figure 8:**
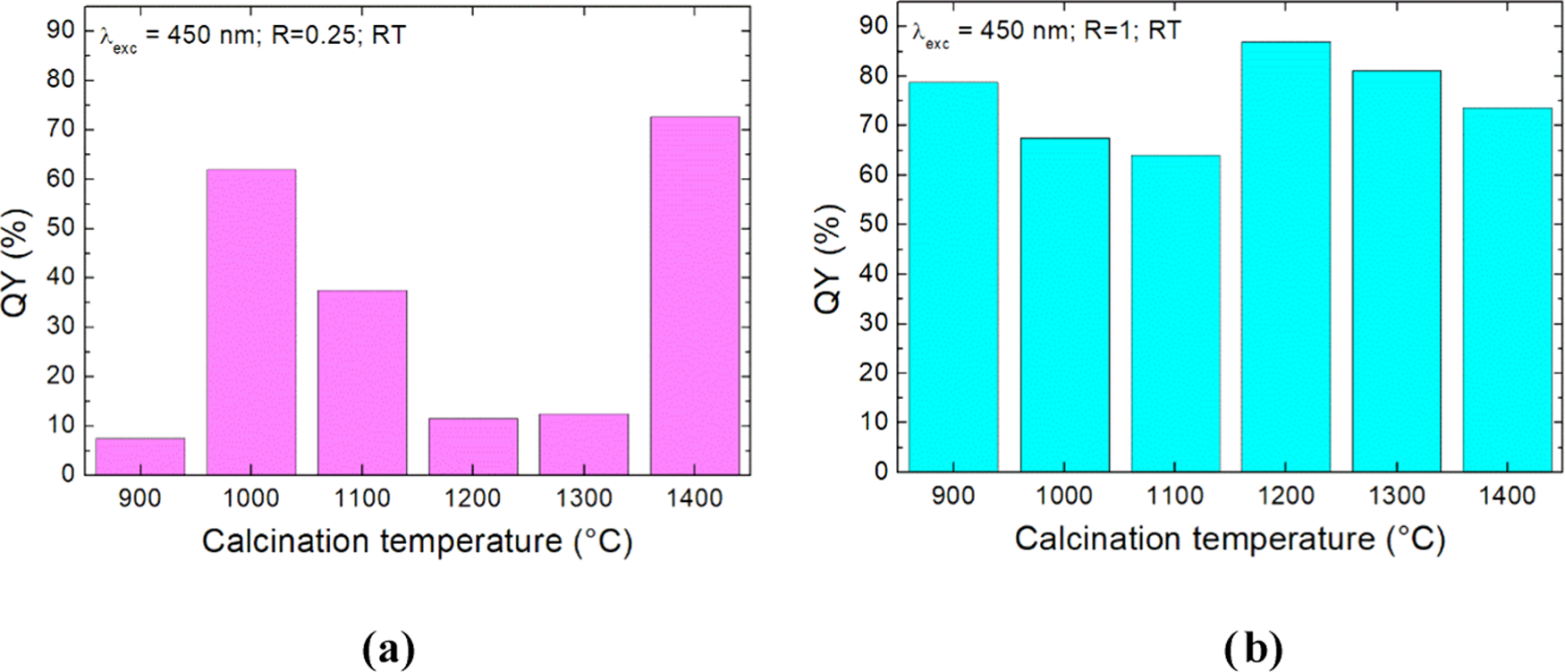
Quantum yields of the YAGG:Ce^3+^,Cr^3+^,Pr^3+^ powder synthesized at *R* = 0.25 (a) and 1 (b).

For both investigated series, quantum yields are very low for excitation at 350 nm (Figure S8). This may be because the total emission is only due to the transfer of energy from Ce to Pr and Cr. In the case of λ_exc_ = 450 nm, the energies of all doped ions exited at the same time and QY are higher. In this case, for the *R* = 0.25 series, no trend is observed with increasing calcination temperature. However, for the *R* = 1 series for the same relation, more stable values of the quantum efficiency are observed with small deviations within the error limit. As we can see from Figure 8, the QY is maximal for an annealing temperature of 1200 °C.

### Thermoluminescence

**Figure 9:**
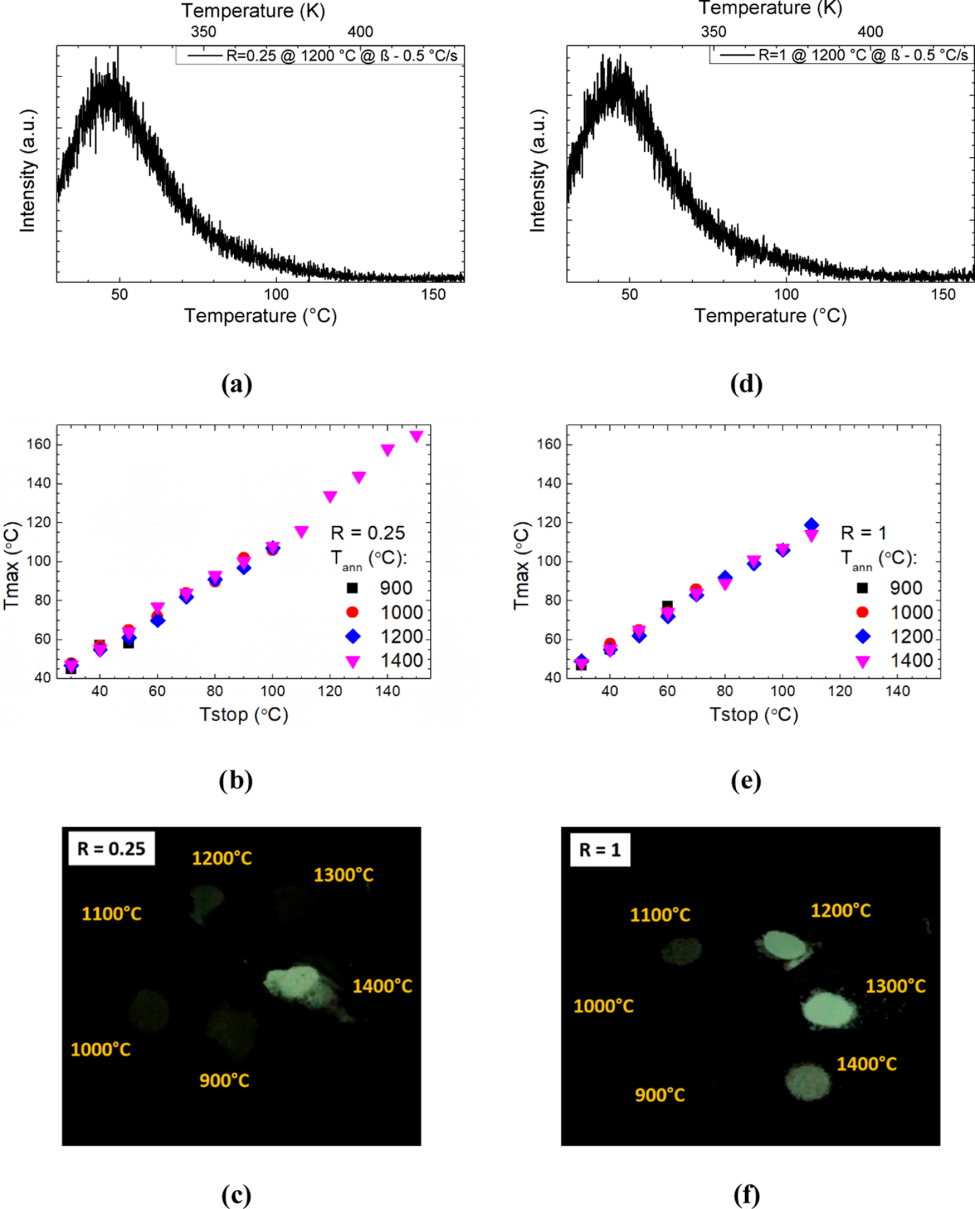
TL curve of the YAGG:Ce^3+^,Cr^3+^,Pr^3+^ powder synthesized at *R* = 0.25 (a) and 1 (d) and calcined at 1200 °C. Graphs of *T*_max_ to *T*_stop_ dependence (b, e), and the photograph of the obtained powder after UV irradiation (c, f).

The TL curves have been acquired to obtain information about the traps in obtained nanophosphors and the effect of urea/metal molar ratios on the trap redistribution. The recorded TL curves (Figures 9 and S9) clearly show the main band with a maximum at ∼47 °C (∼320 K) and the additional band on the high-temperature side, which is not clearly pronounced against the background of the main band with a maximum of ∼87 °C (∼360 K). The general shape of the TL curve is similar to that obtained previously for such structure syntheses with other methods.^30,40^ For both urea/metal ratios (*R*), TL curves are quite symmetrical (except for the band on the high-temperature side), which indicates a homogeneous redistribution of traps with similar energy. Nevertheless, the contribution from the trap component with the TL maximum at around 47 °C is the most pronounced. However, these traps are not clearly separated, which may indicate the free transfer of electrons between them. For a more detailed analysis of the redistribution of traps and the nature of the additional band, a series of TL curves (Figure S10) was registered based on the method of precleaning traps, a so-called *T*_max_–*T*_stop_ method.^45^

Based on the obtained data, plots of *T*_max_ versus *T*_stop_ are drawn and presented in Figure 9b,e for *R* = 0.25 and 1, respectively. The linear nature of the obtained curves with a line of a slope near 1.0 indicates that we have a series of first-order peaks. As the peaks overlap more, the interpretation tends toward the existence of a quasi-continuous distribution of peaks (and, therefore, of trapping centers).^59,60^ As shown in the plots, the final release temperature for the sample synthesized at *R* = 0.25 is 150 °C (for calcination temperature 1400 °C), while for *R* = 1, it is 120 °C. As a result, long-term luminescence is registered only for nanoparticles with an annealing temperature of 1400 °C (Figures 9c and S11a). It should be noted that for samples synthesized at *R* = 0.25, the slope was 0.65 ± 0.3 for nanoparticles annealed at 900 °C and approached 1 with increasing annealing temperature. It was 0.98 ± 0.03 for the sample annealed at 1400 °C. By comparing these data with the PersL of this series (Figures 9c and S11a), we can assume that there is a correlation between the glow and the degree of trap redistribution. In particular, we can expect that with a uniform quasi-continuous distribution of traps, the retrapping process is more uniform and thus contributes to long-term emission, while, for the series of single traps, the detrapping process will be uneven, and the recapture process will increase, resulting in increased losses and reduced afterglow time. In addition, the final release temperature for the sample synthesized at *R* = 0.25 is 150 °C, while for *R* = 1, it is 120 °C (in both cases when calcined at 1400 °C). This means that for *R* = 0.25, there are traps with maxima at higher temperatures. At the same time, the samples synthesized at *R* = 1 show a completely different trend of the slope in terms of trap redistribution based on *T*_max_–*T*_stop_ data analyses. The slope increases from 0.78 ± 0.1 to 0.98 ± 0.02 with increasing calcination temperature from 900 to 1200 °C and then begins to decrease to 0.85 ± 0.02 at 1400 °C, which in turn also correlates with the brightness of PersL (Figures 9f and S11b). Thus, the effect of *R* on the resulting redistribution of traps is worth noting, namely, for *R* = 1, it is possible to obtain nanophosphors with a smaller size of crystallites.

To analyze these traps, in addition to the *T*_max_–*T*_stop_ method, initial rise analyses^61^ were used to determine the energies of each of the recorded curves. As a result, it was found that the activation energies for both series are the same and equal to around 0.75 ± 0.01 eV. Additionally, the analysis of the data using the initial rise method allowed the possibility for us to dedicate a series of traps with an energy of about 0.3 eV for a band from the high-temperature side for both samples. The existence of shallow traps can have an additional impact on the observed effects (PersL), and their nature may be directly related to structural changes, including surface defects.

The similarity of the activation energy for the samples from both series with different results with respect to the afterglow may indicate a nonlinear relationship between the depth of the trap and the efficiency of the glow, as previously reported.^40,62^ By linking these results with the previous analysis of the structure and optical properties, it can be concluded with a high probability that despite traps with similar energy values, the intensity and duration of PersL may be influenced by other factors. In particular, morphology, phase change, redox reactions of the dopant ions, changes due to clustering of such dopants, and so on. However, this should be further established.

## Conclusions

The synthesis of YAGG:Ce^3+^,Cr^3+^,Pr^3+^ crystallites with sizes starting from 45 nm by the urea glass route method (UGR) is successfully realized. UGR can be considered a more ecological, inexpensive, one-pot method with high yields, and therefore, it is suitable for large-scale production. The UGR method is relatively easy to carry out and gives results similar to other methods we have previously tested. The ratio of urea/metal salts, *R*, influences the formation of the gel-like, and for this study, we selected two systems at *R* = 0.25 and 1. The gel-like prepared at *R* = 0.5 was not selected because of a few differences in behavior observed between them. The two gel-like substances were then treated at 900, 1000, 1100, 1200, 1300, and 1400 °C for 1 h. From the structural and morphological point of view, there are no substantial differences in the cell parameters, crystallite size, and particle size with *R*. However, at the same *R*, the increase in size occurs with increasing temperature. At all temperatures, the composition of the powders respects the nominal ratio between atoms in the powders. Only some samples, treated at higher temperatures, showed efficient persistent luminescence. Regardless of synthesis parameters, we obtain nanopowders with long-term emission, which is associated with the presence of a series of first-order quasi-continuous distribution traps, which, in terms of applications, are important and contribute new knowledge. In potential biological applications and also in the field of luminescent labeling, not always duration and intensity are the most important criterion for their suitability. By changing the parameter *R*, it is possible to achieve a more uniform distribution of quasi-continuous traps, which in turn has a positive effect on obtaining PersL from phosphors in the nanoscale range. Additional maxima at the shoulder of high temperatures with an energy of around 0.3 eV are detected. This may be due to the presence of traps associated with structural imperfections in particular to the heterogeneous redistribution of dopant ions on the surface of the obtained nanophosphors.

## Supporting Information Available

TEM micrographs of YAGG:Ce^3+^,Cr^3+^,Pr^3+^ powders calcinated at 1400 °C for 1 h (Figure S1); selected area electron diffraction (SAED) pattern and EDS analysis (Figure S2); photoluminescence excitation spectra as a function of calcination temperature for *R* = 0.25 and 1 (Figure S3); photoluminescence spectra of YAGG:Ce^3+^,Cr^3+^,Pr^3+^ at λ_exc_ = 350 nm (Figure S4); photoluminescence lifetimes of YAGG:Ce^3+^,Cr^3+^,Pr^3+^ as a function of calcination temperature at λ_exc_ = 350 (Figure S5); photoluminescence lifetimes of YAGG:Ce^3+^,Cr^3+^,Pr^3+^ as a function of calcination temperature at λ_exc_ = 450 nm (Figure S6); average lifetime as a function of the calcination temperature and excitation and detection wavelengths (Figure S7); quantum yields of YAGG:Ce^3+^,Cr^3+^,Pr^3+^ at λ_exc_ = 350 nm (Figure S8); thermoluminescence curves of YAGG:Ce^3+^,Cr^3+^,Pr^3+^ (Figure S9); thermoluminescence glow curves registered during *T*_max_–*T*_stop_ experiments (Figure S10); and persistent luminescence decay curve after irradiation by a blue laser diode λ_irr_ – 450 nm (Figure S11)(PDF)
