# Supplementary material for: Urea Glass Route as a Way to Optimize YAGG:Ce3+,Cr3+,Pr3+ Nanocrystals for Persistent Luminescence Applications
Source: Langmuir. 2022 Sep 13;38(38):11539–49. doi: 10.1021/acs.langmuir.2c00687 (PMC9520973; doi:10.1021/acs.langmuir.2c00687)
Supplement: Supplementary file 1 — la2c00687_si_001.pdf [file la2c00687_si_001.pdf]

Supplementary Information

Urea-glass route as a way to optimize $Y_3Al_2Ga_3O_{12}:Ce^{3+},Cr^{3+},Pr^{3+}$ nanocrystals for persistent luminescence applications

Vitalii Boiko[a*], Maria Luisa Saladino[b*], Francesco Armetta[b] , Federica Ursi[b], Marta Markowska[a], Karina Grzeszkiewicz[a], Cecilia Mortalò[c], Cristina Leonelli[d], Dariusz Hreniak[a]

[a] *Institute of Low Temperature and Structure Research, Polish Academy of Sciences, ul. Okólna 2, PL-50-422 Wrocław, Poland*

[b] *Department of Biological, Chemical and Pharmaceutical Sciences and Technologies (STEBICEF) and INSTM UdR - Palermo, Università di Palermo, Viale delle Scienze Bld. 17, IT-90128 Palermo, Italy*

[c] *Institute of Condensed Matter Chemistry and Energy Technologies (ICMATE), National Research Council of Italy, Corso Stati Uniti 4, IT-35127 Padova, Italy*

[d] *Department of Engineering "Enzo Ferrari", University of Modena and Reggio Emilia, Via Pietro Vivarelli 10, IT-41125 Modena, Italy*

**corresponding authors: marialuisa.saladino@unipa.it, v.boiko@intibs.pl*

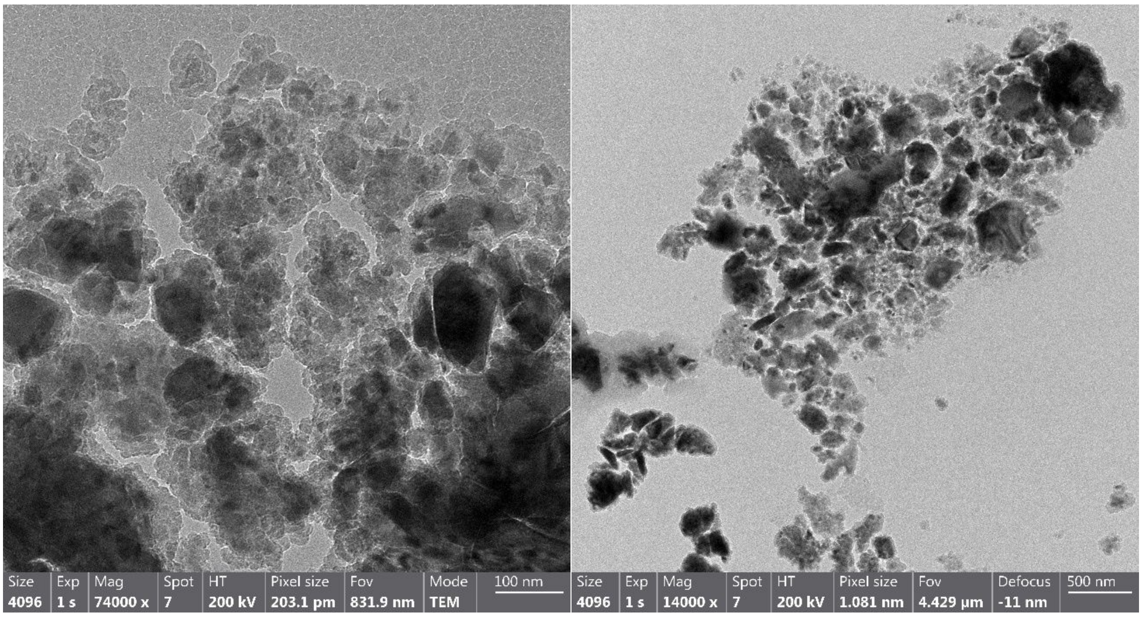

Figure S1. TEM micrographs of YAGG:Ce^{3+},Cr^{3+},Pr^{3+} powders calcinated at 1400 °C for 1 h.

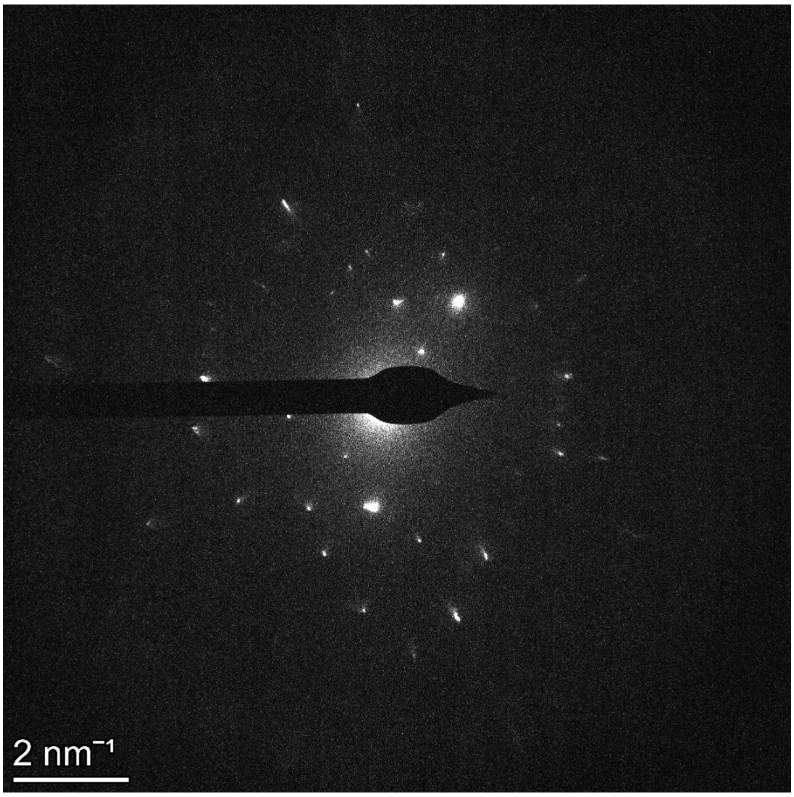

a

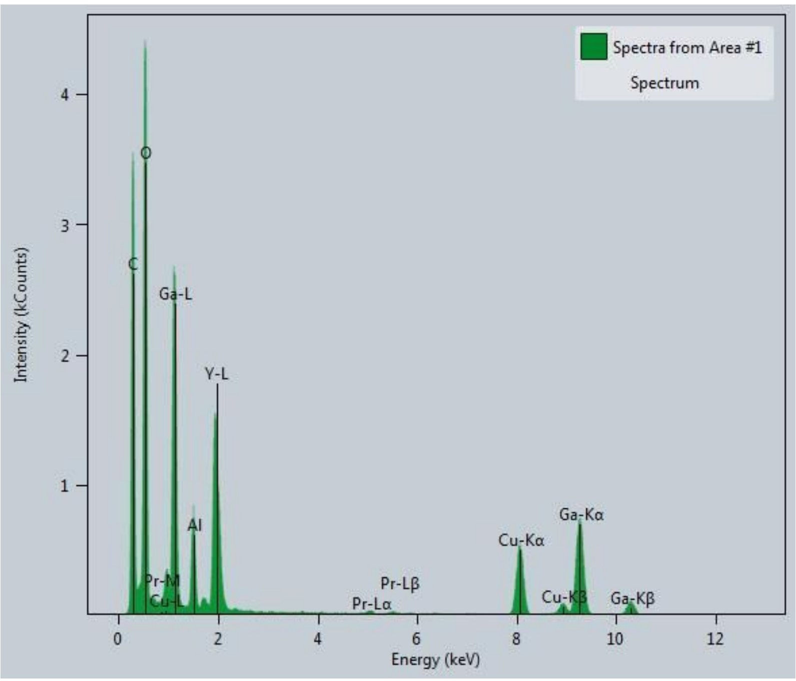

b

Figure S2. Selected Area Electron Diffraction-SAED pattern (a) and EDS analysis (b) of the YAGG:Ce^{3+},Cr^{3+}, Pr^{3+} sample synthetized at R=1 and calcined at 1000°C for 1 h. SAED pattern is typical of a polycrystalline powder. Please notice the peak of Cu in the EDS spectra is due to the copper grid holding the specimen.

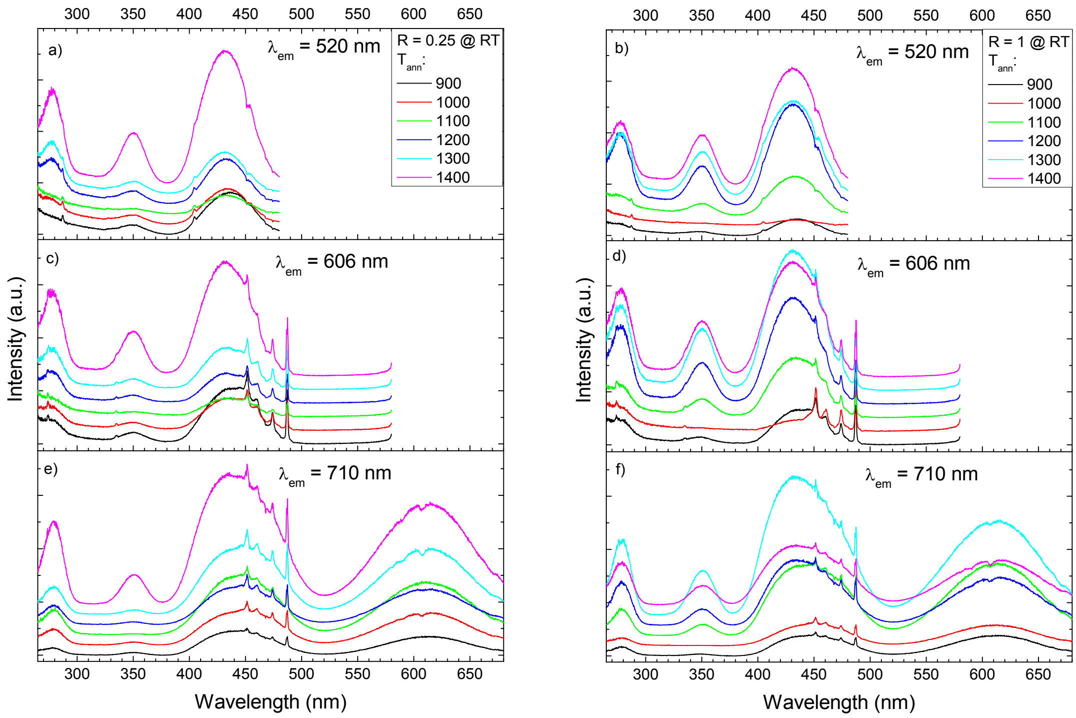

Figure S3. Photoluminescence excitation spectra as a function of calcination temperature were observed at 520 nm (a) and (b) at 606 nm (c) and (d) and at 710 nm (e) and (f), for R=0.25 left panel and R=1 right panel.

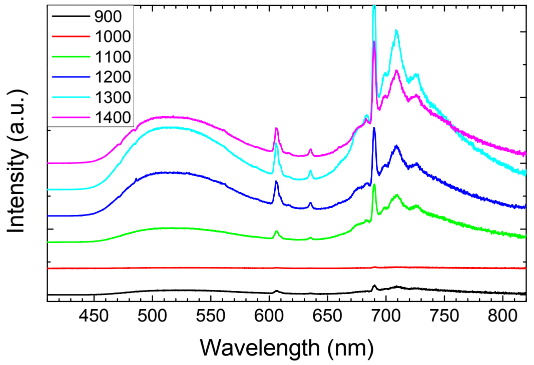

a)

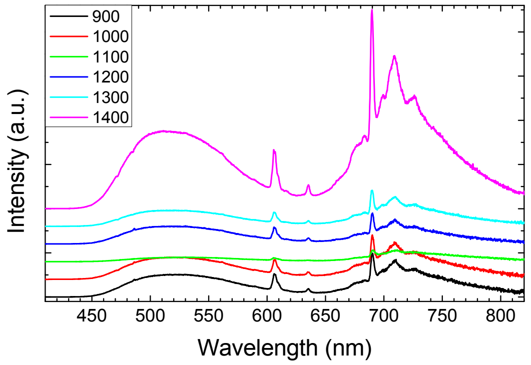

b)

Figure S4. Photoluminescence spectra of YAGG:Ce^{3+},Cr^{3+},Pr^{3+} synthesised at R = 0.25 (a) and 1 (b). λ_{exc} = 350 nm, RT

Photoluminescence decay time curves were detected for specific emission bands Ce^{3+} (520 nm) Pr^{3+} (606 nm) and Cr^{3+} (710 nm) after excitation at 350 nm (specific for Ce^{3+}). For Pr^{3+} and Cr^{3+} emission, this excitation is not out of the absorption bands and the detected emission is a result of the energy transfer process from Ce^{3+}.

**R = 0.25**	**R = 1**
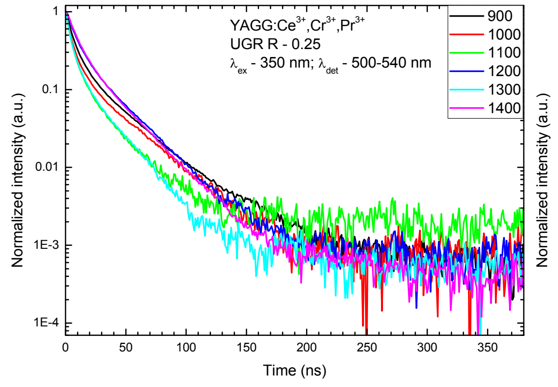	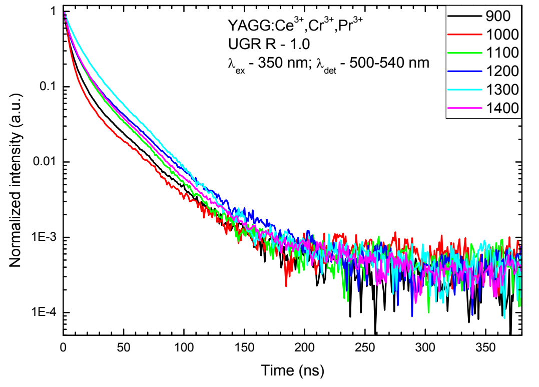
**Ce^{3+}**	
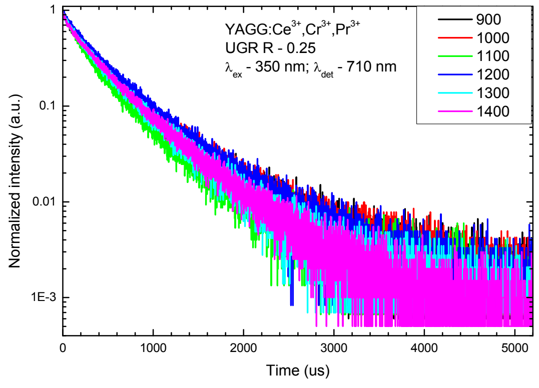	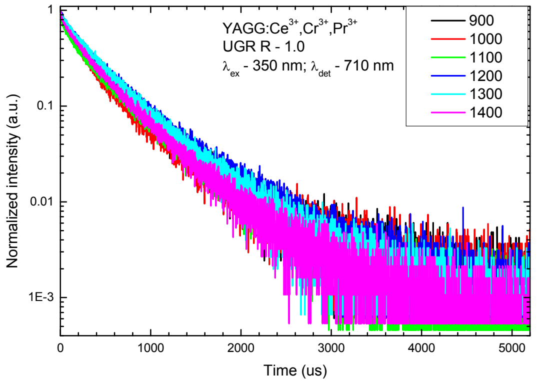

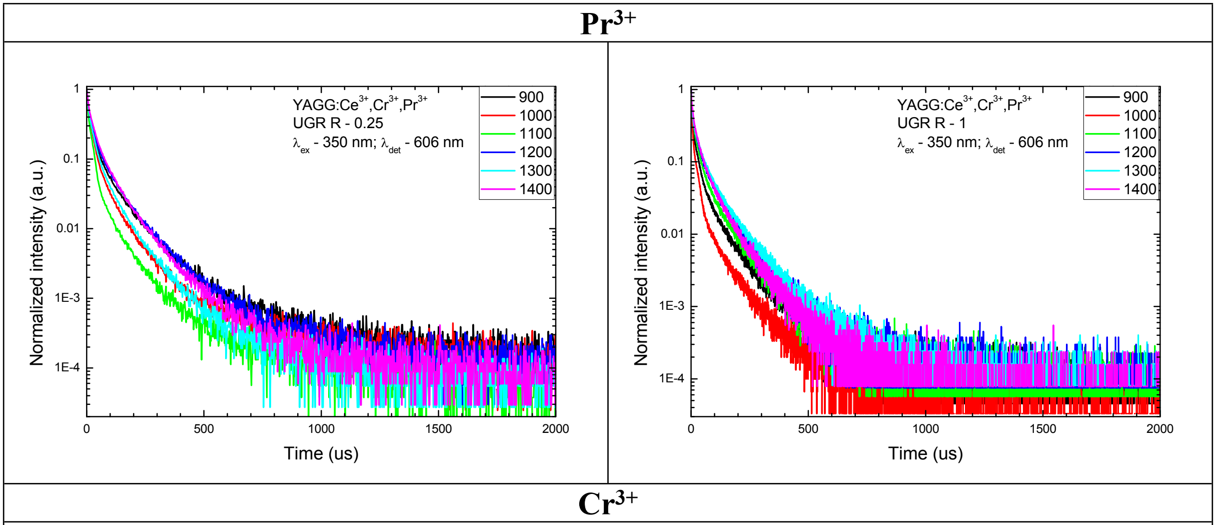

Figure S5. Photoluminescence lifetimes of YAGG:Ce^{3+},Cr^{3+},Pr^{3+} synthesised at R = 0.25 (left panel) and 1 (right panel) as a function of calcination temperature. λ_{exc} = 350 nm at room temperature.

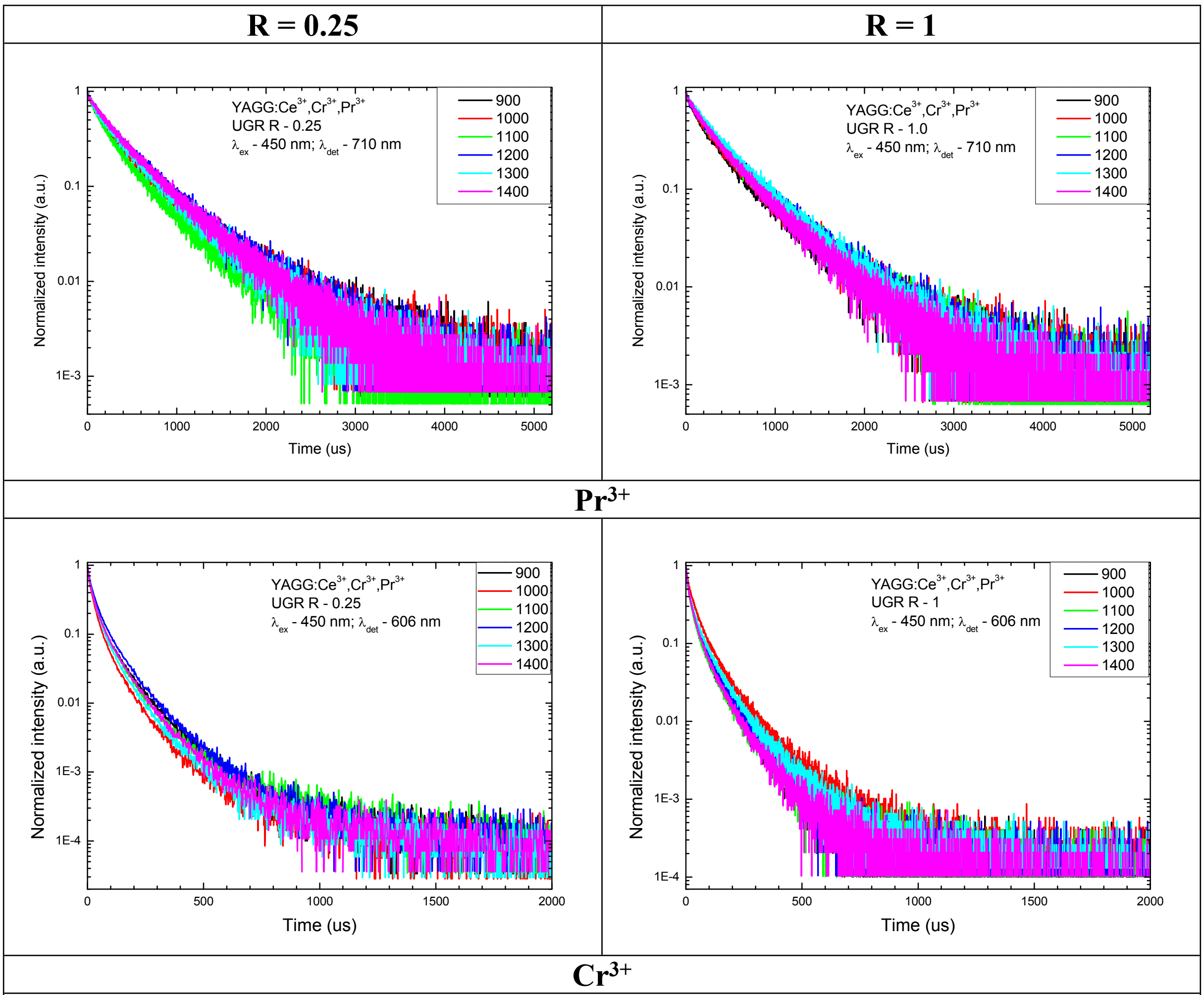

Figure S6. Photoluminescence lifetimes of YAGG:Ce^{3+},Cr^{3+},Pr^{3+} synthesised at R = 0.25 (left panel) and 1 (right panel) as a function of calcination temperature. λ_{exc} = 450 nm at room temperature.

Based on the acquired decay curve the average decay time for each curve is calculated and placed in the table below.

Excitation 350	Excitation 450
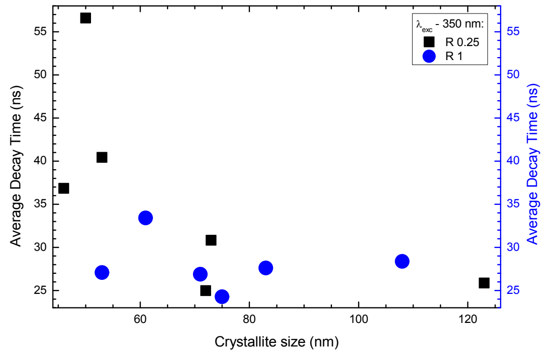	
**Ce^{3+}**	
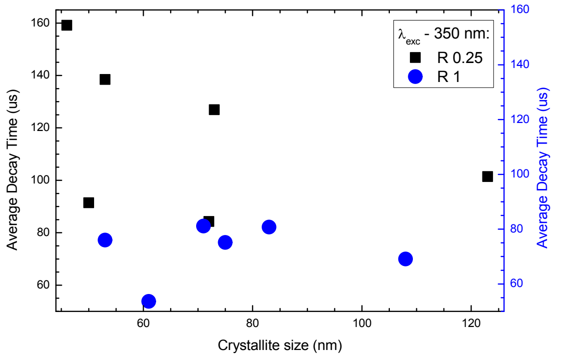	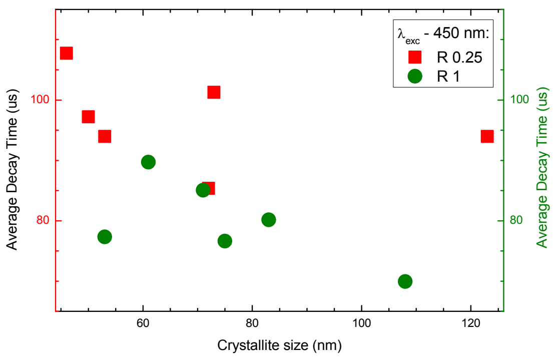
**Pr^{3+}**	
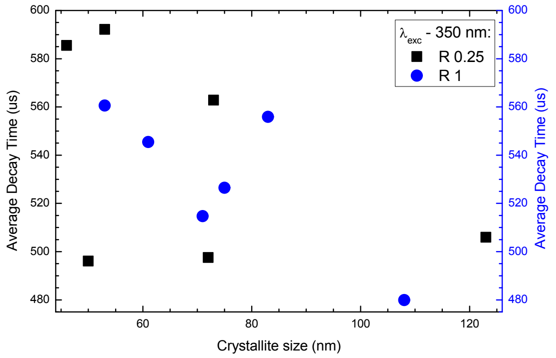	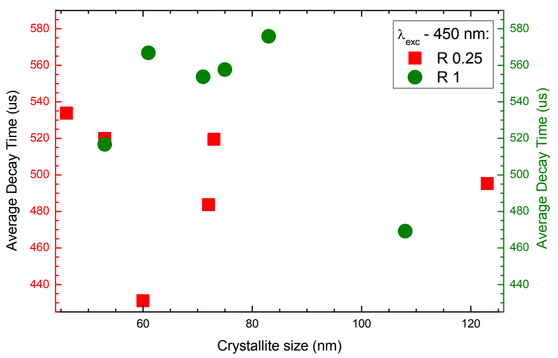
**Cr^{3+}**	

Figure S7. Average lifetime as a function of the calcination temperature excitation and detection wavelength.

The external QY for all samples was detected after excitation for both 350 and 450 nm with an integrating sphere and followed calculated using the formula:

$$QY = \frac{A_{Sem} - A_{Rem}}{A_{Rexc} - A_{Sexc}} \quad (1)$$

where A_{Sem} – area of the sample emission; A_{Rem} – area of the reference emission (matrix without doped ions) and A_{Rexc} and A_{Sexc} – an area of the excitation band of the sample and reference, respectively.

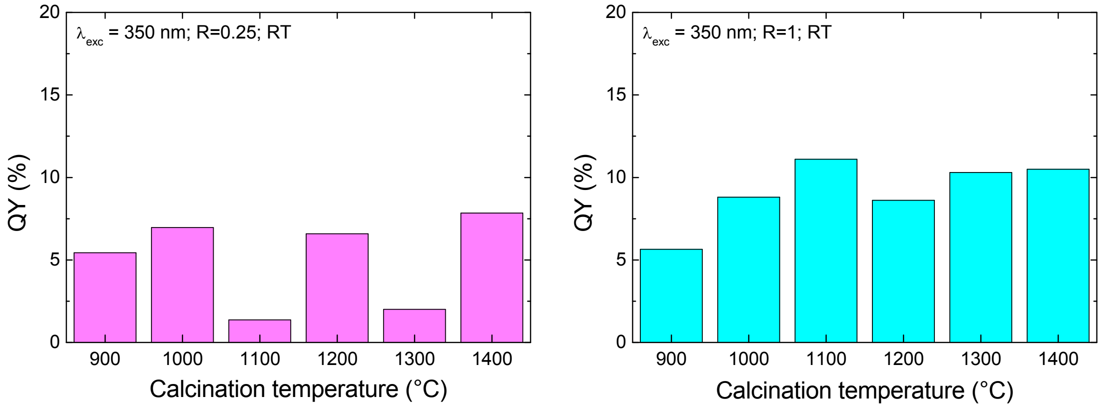

Figure S8. Quantum yields of YAGG:Ce^{3+},Cr^{3+},Pr^{3+} obtained at R=0.25 (left) and R=1 (right) after excitation at 350 nm.

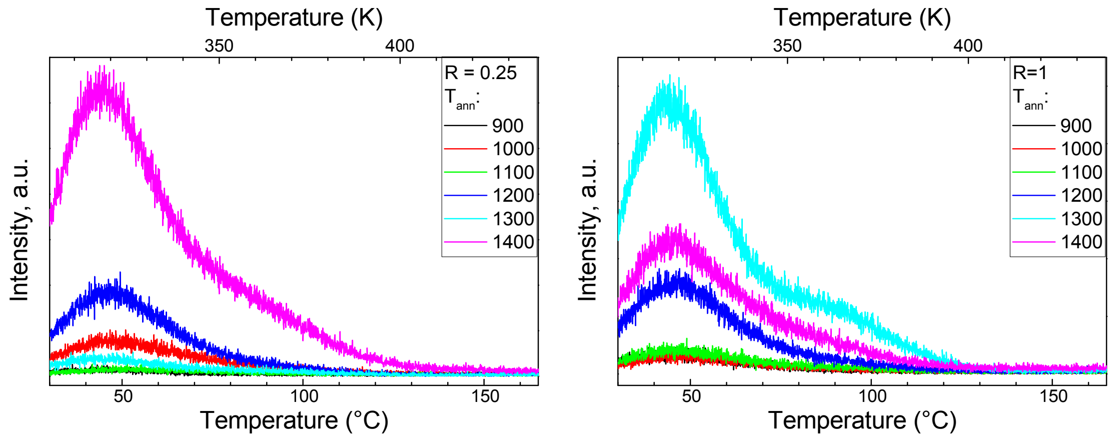

a) b)

Figure S9. Thermoluminescence curves of YAGG:Ce^{3+},Cr^{3+},Pr^{3+} obtained at R=0.25 (a) and R=1 (b). Irradiation at 450 nm for 5 min, β – 0.5 °C/s.

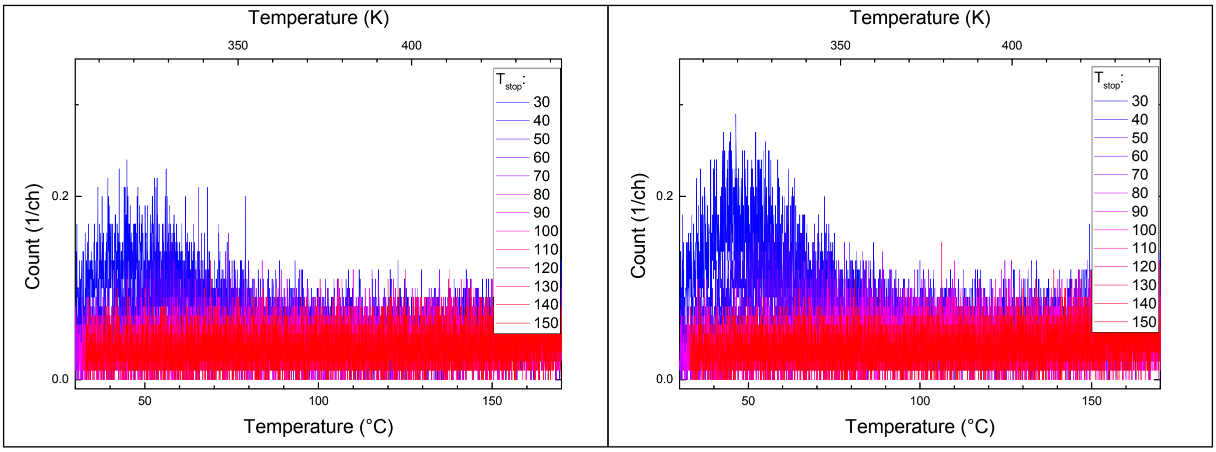

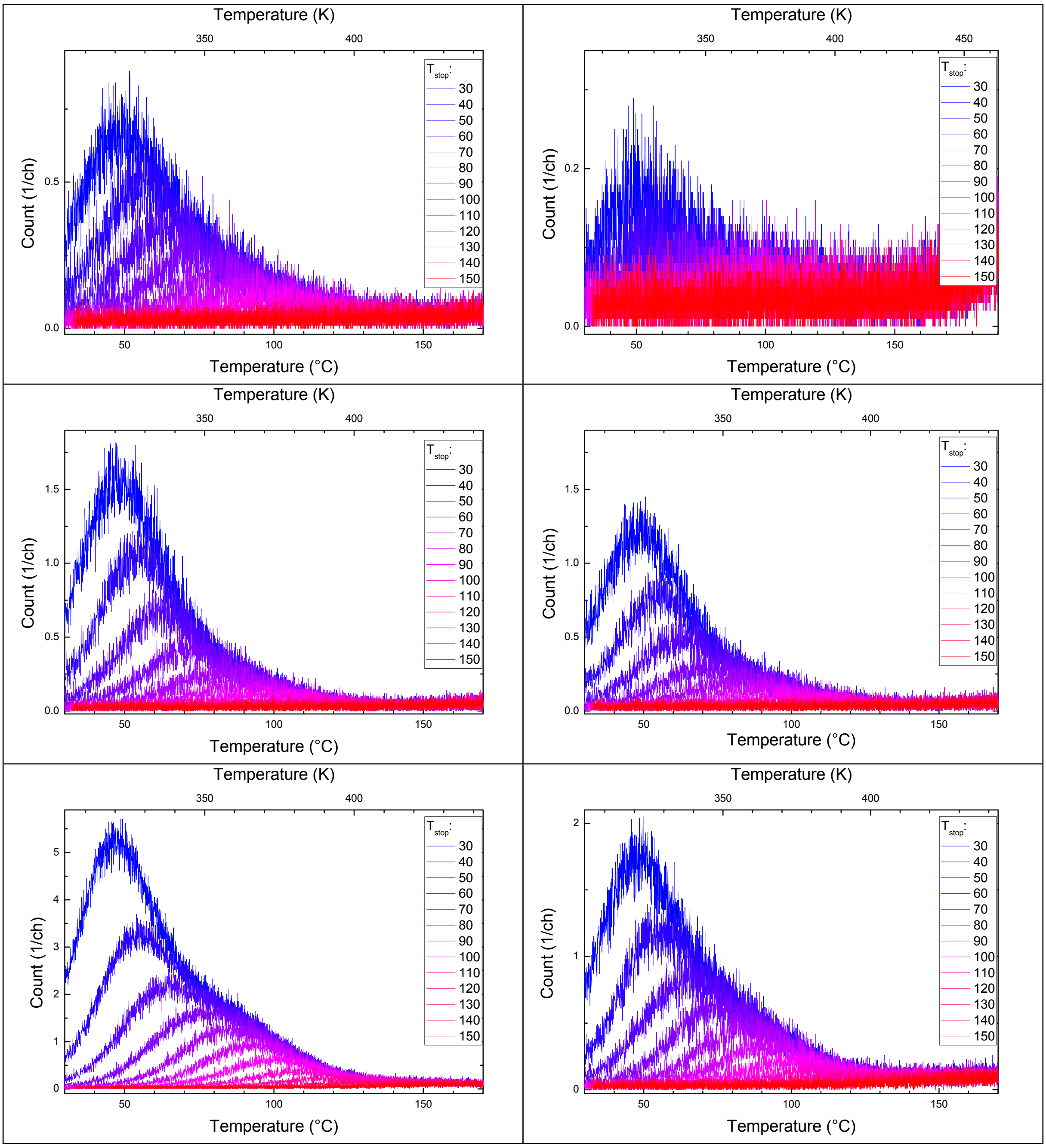

Figure S10. Thermoluminescence glow curves registered during T_{max}-T_{stop} experiments. Samples were charged with blue laser diode λ_{exc} – 450 nm.

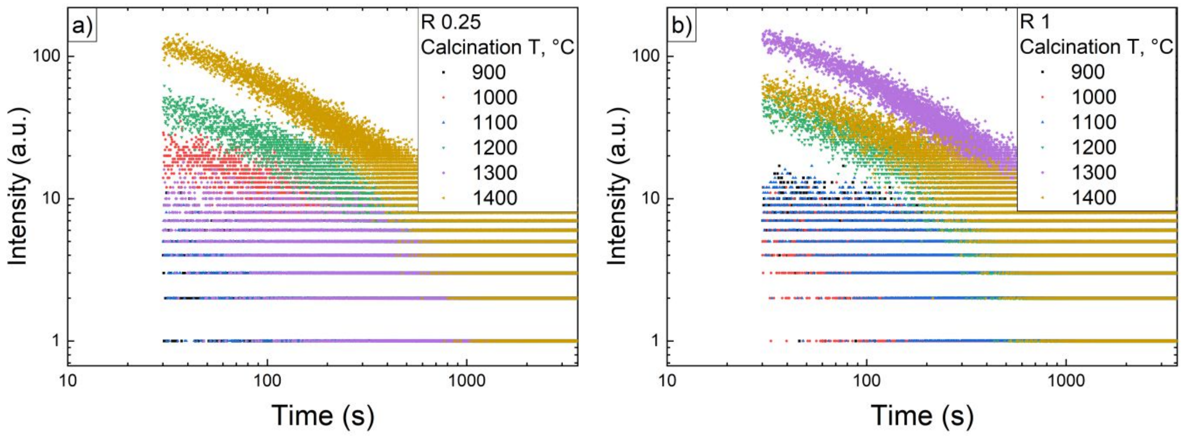

Figure S11. Persistent luminescence decay curve after irradiation by blue laser diode λ_{irr} – 450 nm for 5 min and brake before detection 1 min.
